# Creative Forces^®^ Creative Arts Café: a theory-based creative performance framework for military-connected populations with traumatic brain injury and posttraumatic stress disorder

**DOI:** 10.3389/fpsyt.2026.1734583

**Published:** 2026-03-26

**Authors:** Rebecca Vaudreuil, Abigail Palmer, Nicole Moret, Elizabeth K Freeman, Paul Sargent, Peter Buotte, Gioia Chilton

**Affiliations:** 1 Henry M Jackson Foundation for the Advancement of Military Medicine Inc, Bethesda, MD, United States; 2 Abbie Palmer Music, Hedgesville, WV, United States; 3 Daniel K. Inouye Graduate School of Nursing, Uniformed Services University of the Health Sciences, Bethesda, MD, United States; 4 National Intrepid Center of Excellence, Walter Reed National Military Medical Center, Bethesda, MD, United States; 5 Cloud Nine Clinical Consultants, Encinitas, CA, United States; 6 Intrepid Spirit Center, Carl R. Darnall Army Medical Center, Fort Hood, TX, United States; 7 Intrepid Spirit Center, Alexander T Augusta Military Medical Center, Fort Belvoir, VA, United States

**Keywords:** creative arts therapies, Creative Forces^®^, creative performance, interdisciplinary treatment, military personnel, postraumatic stress disorder, traumatic brain injury, veterans

## Abstract

Creative arts therapies integrated into interdisciplinary care supports treatment optimization of military-connected individuals with traumatic brain injury, posttraumatic stress disorder, and related health concerns. This paper explores the use of creative performance in interdisciplinary treatment that includes art therapy, dance/movement therapy, and/or music therapy through literature review and discusses implications for physiological, psychological, and psychosocial rehabilitation. It presents an applied model – the Creative Forces Creative Arts Café (CF-CAC) – used with patients receiving creative arts therapies as standard care at military treatment facilities across the United States. A theoretically informed CF-CAC framework provides a structured approach to utilizing creative performance as a clinical tool to support treatment goals and includes guidance for preparation, implementation, and post-experience processing. Core concepts of creative performance that underpin the CF-CAC are informed by creative arts therapies research, social health and human performance models, neurologic theory, and strengths-based approaches that encourage patient engagement, increase treatment adherence, support goal attainment, invite staff collaboration, and promote familial and community engagement. Considerations for facilitating the CF-CAC using virtual platforms to meet the continued demand for tele-connection are discussed. Standardized health measures for formally evaluating the CF-CAC to inform long-term sustainable gains from engagement in creative performance are recommended.

## 1 Introduction

Interdisciplinary treatment models are gaining traction as an approach to care delivery in military, veteran, and civilian healthcare systems due to their beneficial impact on patient outcomes (1–3). Over the past decade, creative arts therapies (CATx) have been integrated as a core service alongside traditional rehabilitation and behavioral health disciplines at United States (U.S.) military and veteran treatment facilities (4–6). Through these efforts, emerging research has reported positive results from creative arts engagement in clinical (e.g., military and VA treatment facilities) and non-clinical (e.g., community-based) settings as part of patients’ rehabilitation pathways, overall treatment experiences, and reintegration efforts (7, 8). Preliminary findings indicate that the intentional use of creative performance coupled with CATx interventions is a promising method that supports clinical skill generalization, socialization, and reintegration for military-connected individuals, which include active-duty service members and veterans as well as their families and caregivers (9, 10).

Drawing from CATx literature (11, 12), integrated human performance models (13), and social health frameworks (14), this paper highlights the use of creative performance to bolster clinical outcomes for military-connected individuals receiving interdisciplinary treatment for traumatic brain injury (TBI) and posttraumatic stress disorder (PTSD) that includes CATx as a core rehabilitative service. Since 2000, over 500,000 service members have been diagnosed with TBI and over 80% of these cases are classified as mild (15). Additionally, 29% of military personnel who served in Operations Iraqi Freedom and Enduring Freedom experience PTSD (16). Longitudinal studies report that PTSD and other psychological variables prevalent in military populations may impede TBI recovery, particularly when the injury is mild, for example, a concussion (17). This further complicates the clinical landscape of military personnel who are treated for co-morbid TBI and PTSD, which share symptomatology of anxiety, depression, sleep disruption, substance use, hypervigilance, shame, isolation, and suicidality (4).

This paper discusses the impact of integrating creative performance into art therapy, dance/movement therapy, and/or music therapy treatment delivered in military healthcare with patients diagnosed with TBI, PTSD, and/or related sequelae. Creative performance is operationally defined herein as “*the intentional use of the arts to cultivate and motivate action orientation towards accomplishing goal-based tasks combined with behavioral, neurological, socioemotional, familial, occupational, and functional skills training”*. A theoretically informed framework and clinical examples contextualize patient benefits, which include increased treatment adherence, support of rehabilitation goals, and promoting familial, social, and community engagement. Safety concerns related to patient participation are also addressed.

There are few publications that report music therapists’ use of creative performance with military-connected individuals (4, 11, 12); however, this is the first to detail the depth and breadth of implementation processes, describe utility across CATx services, and demonstrate integration into interdisciplinary treatment. Further, this paper contributes to existing literature on the topic of creative performance in CATx treatment with U.S. military-connected populations, which is limited.

### 1.1 Introducing an integrated creative performance model

The Creative Forces Creative Arts Café (CF-CAC) is a performance platform that provides opportunities for patients, families, and staff to connect through artistic and expressive outlets and supports patients in sharing clinical products from CATx treatment (e.g., musical pieces, artwork, dances) with wider audiences (4). A publication by Vaudreuil et al. (11) provided case examples that indicated positive outcomes for service members with TBI and PTSD who engaged in CF-CAC performance as part of their music therapy treatment. One example reported how a U.S. Army Sergeant with severe TBI used creative performance as a catalyst to motivate therapeutic progress, apply skill generalization, and support the transition from active duty to veteran status. Another example illustrated a U.S. Marine with chronic combat-related PTSD who used creative performance to help transform distress responses, reframe his trauma narrative, and improve spousal communication.

CATx interventions encourage the development of coping mechanisms through creating a structured environment wherein strong therapeutic alliances can form (18, 19). Using creative performance as a clinical tool in CATx treatment can support rehabilitative processes that lead to outcomes such as experiencing hope and gratification in recovery from traumatic experiences through meaning-making (20), enabling positive reframing (21), and reducing symptoms associated with PTSD and TBI such as pain, stress, hypervigilance (22, 23), and disrupted sleep (4). Further, this dynamic treatment approach is highly valued by patients, their families, and interdisciplinary team members in military healthcare settings (7).

### 1.2 History of the CF-CAC

The CF-CAC was initiated in 2016 by the CATx program at the National Intrepid Center of Excellence, a specialty clinic at Walter Reed National Military Medical Center that provides interdisciplinary patient-centered care for service members with TBI, PTSD, and related health concerns (4, 11). The CF-CAC was designed by creative arts therapists (CATs) in alignment with the clinic’s integrated treatment model (e.g., patient-centered, interdisciplinary, continuity of care) aimed at bolstering the patient experience by traversing a continuum from clinical CATx treatment to community arts engagement (24). Since its inception, the CF-CAC has been replicated by CATs working at military clinics networked with the National Intrepid Center of Excellence that operate as part of the Defense Intrepid Network for TBI and Brain Health and include CATx as standard care (25). This multifaceted model of creative performance is well-positioned to accompany interdisciplinary treatment that addresses cognitive, behavioral, and socioemotional concerns through competency training and cultivating experiences that expand patients’ capacities across functional realms (26).

### 1.3 Interagency partnership support

**Figure 1:**
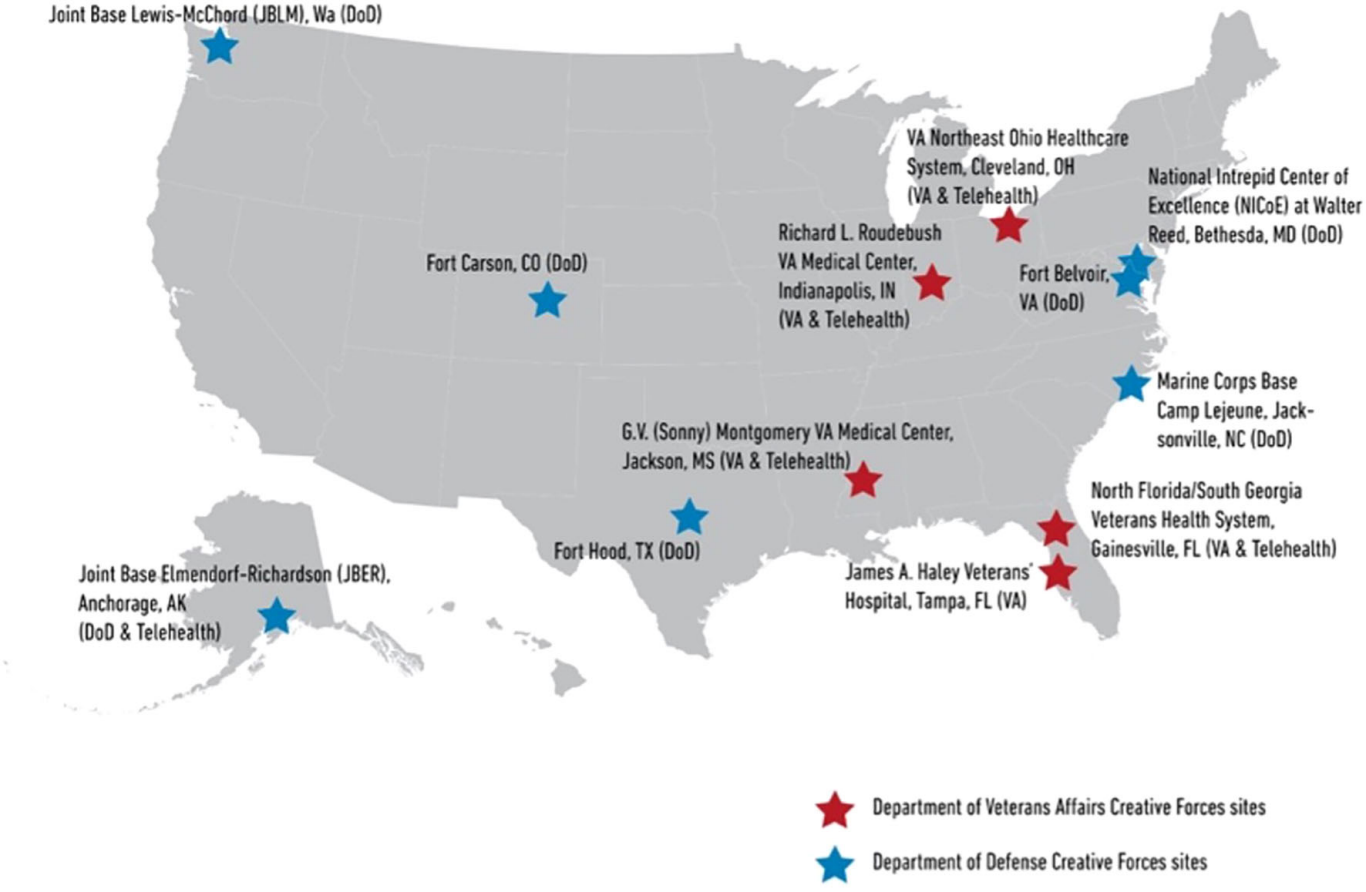
Creative Forces site locations. Source: reproduced from National Endowment for the Arts (28), with permission.

The establishment and continued advancement of creative performance into CATx treatment is possible through interagency partnerships between federal and non-governmental organizations in the U.S. that extend across systems levels – from local to national and from the clinic to the community. A partnership between the National Endowment for the Arts (NEA) and the U.S. Departments of Defense (DoD) and Veterans Affairs (VA), Creative Forces^®^: NEA Military Healing Arts Network (CF), seeks to improve the health, well-being, and quality of life for military and veteran populations exposed to trauma, as well as their families and caregivers (27). CF supports CATx clinical programming, specifically art therapy, dance/movement therapy, and music therapy as part of interdisciplinary treatment programs at DoD facilities and VA medical centers throughout the U.S. As of 2025, CF CATx programs operate at seven DoD and five VA clinical sites, as shown in **Figure 1**, and include a telehealth component to reach rural, remote, and home-bound individuals, communities, and widely dispersed military commands (6, 29).

CATs working at these sites develop goal-based treatment plans on a continuum that supports rehabilitation goals, trains skill-building, and encourages patients to apply creative strategies introduced in clinical sessions in other environments they routinely navigate in their daily lives (e.g., home, work, community). This is aligned with CF efforts to increase access to community arts opportunities for military-connected individuals supported through a national grant program that aims to promote creative expression, build social connections, and improve resiliency (30). Further, CF invests in researching and disseminating content that demonstrates the value and impact of CATx treatment and community arts engagement to advance knowledge, leverage subject-matter expertise, and promote informed use of arts-based practices with military-connected individuals and communities (31).

### 1.4 Creative performance on a clinic-to-community continuum

Oftentimes, when active-duty service members engage in treatment, they continue to maintain military requirements and responsibilities. Similarly, veterans navigate treatment with consideration to work schedules. If a service member or veteran is solely focused on their role as a patient, this may become a barrier to meeting occupational demands, reintegration efforts, and other obligations. Conversely, if a service member or veteran has difficulty acknowledging the need for treatment, this could prevent them from seeking necessary care. According to Kass et al., “establishing a strong clinic to community continuum is critical in supporting transition as patients leave treatment and addresses the needs of individuals who may not seek access to traditional clinical care” (2022, p. 99). The CF-CAC model supports this continuum by creating environments in which CATs can collaborate with local artists, arts organizations, and military agencies to share information, build reciprocally purposeful relationships, and design culturally informed community arts engagement opportunities for military-connected populations (32).

**Figure 2:**
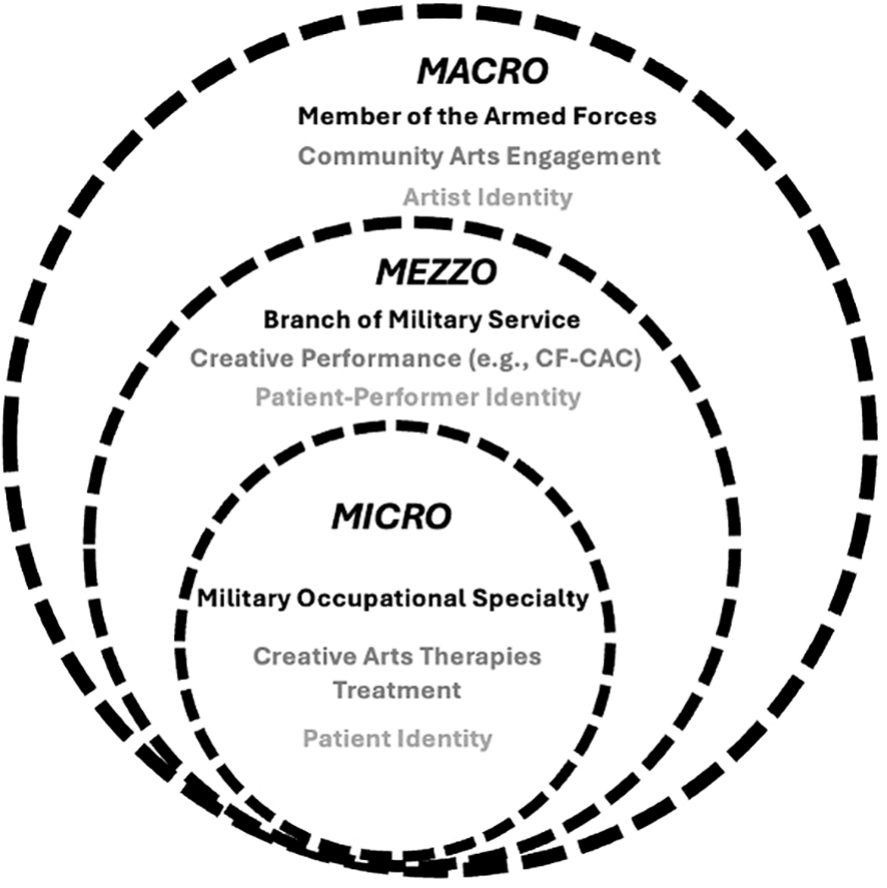
Systems levels of military service, CATx treatment, and creative engagement.

Just as career professionals often build strong identities rooted in their work, military-connected individuals experience this threefold: (1) within their military occupational specialty (micro), (2) as a member of a certain military branch (mezzo), and (3) as a member of the armed forces-at-large (macro). Borrowing from social work’s person-in-environment model (33), **Figure 2** depicts interconnectivity of corresponding military and rehabilitative identities as patients progress through CATx treatment and demonstrates how creative performance can serve as a transitional experience between clinical and community-based arts engagement. This continuum supports patients in amplifying their sense of self, identities, and cultures within and beyond medical or military scopes by becoming more active and creatively engaged members of their greater communities (34, 35), which is essential for priming post-military service reintegration efforts (12).

## 2 Creative performance context: foundational concepts and theoretical frameworks

Engagement in creative performance provides time, space, and structure for military-connected individuals to process identity shifts that commonly occur throughout the rehabilitation process including acclimating to new abilities and accepting variances in functioning (e.g., past to current self), evolving from a novice to an experienced performer (e.g., CF-CAC engagement during treatment), and transitioning from active-duty to veteran status (36). When patients engage in creative performance, CATs reinforce tenets of creative expression, self-acceptance, improvisation, spontaneity, and emphasis on the therapeutic process over product while also integrating military rituals to bolster personal and cultural healing (37–39).

### 2.1 Creative performance in military identity, culture, and rituals

The use of rituals to facilitate identity shifts is common across cultures. Some rituals include rites of passage, in which one moves through liminal, transitional space to shift from one’s former identity to an as-yet-unknown future self (40). Military culture, which is the shared identity, values, practices, and beliefs of military-connected individuals, employs a range of formal and informal rituals that serve as rites of passage (41, 42). Rituals that establish and/or reinforce military positioning may vary in specificity by branch, but are generally realized through promotion and retirement ceremonies, deployment and homecoming celebrations, military balls, presenting the colors, and events of military recognition and remembrance. Within these traditions wherein military identity manifests, a deeper psychological journey may exist (43).

The CF-CAC model uniquely supports identity shifts and rehabilitation transitions through rituals that symbolize treatment progress and completion. For example, creative performance integrated into clinic ‘graduations’ acknowledges treatment completion while simultaneously providing closure to the therapeutic rapport developed within the patient’s clinical process (44). Patients may engage in individual and/or group performances to demonstrate products created in CATx treatment (e.g., songs, dances, artwork) that reflect their recovery process. Additionally, members of the interdisciplinary team may speak about patients’ successes in treatment. The graduations, which mirror military ceremonial traditions, serve as strengths-based rites of passage by recognizing and validating patients’ work in their rehabilitation and setting the stage to continue their creative journeys whether they are returning to active duty or transitioning out of military service (11).

### 2.2 Creative performance for neurologic rehabilitation

Creative performance integrates multiple modes of sensory-based communication to convey meaning and purpose, which are essential components of recovery (12). Theoretical synthesis is needed to underpin the multimodality of the arts, which activate complex neurologic processes to engage motor, visual, and spatial functioning on a biopsychosocial level (45–47). CATx interventions that support neurorehabilitation can be broadly grouped into those that target restoration of function and those that train compensation of deficits, which although distinct may be coordinated to promote optimal rehabilitative benefit. Restoration of function is pursued to the greatest degree possible and compensatory strategies are often applied to support restorative processes, which can continue to support functions even once maximum benefit has been reached (7). For decades, CATx treatment has been identified as a mechanism for functional restoration and performance can play a critical component in recovery. For example, Field and colleagues (1954) used music to motivate patients with TBI to execute targeted movements through instrumental music performance. With repetition, the task became progressively easier, demonstrating the adaptation of nerves to restore previously lost functions. The opportunity for pairing receptive and expressive activities that enhance neurocircuitry by forming and reinforcing neural pathways through repetition (48) is illustrated by the concept of “cells that fire together wire together” (49, p.64).

Further, converging evidence from objective studies on the effect of music intervention on cognitive impairment show that changes in the brain can be detected through spatial (e.g., fMRI) and temporal (e.g., EEG) relationships, as well as cognitive changes (e.g., traditional neurocognitive testing) (50). The process of creating musical, movement-based, and artistic works requires the patient to be purposefully and wholly engaged, and performance-based neurorehabilitation supported by CATx treatment provides multimodal activation of the brain (51–53). Dynamic CATx interventions coupled with creative performance aimed at achieving neurological gains for military-connected individuals can progressively challenge patients, particularly those with TBI and PTSD, in a way that supports complex neurorehabilitation through gratifying processes (20).

### 2.3 Creative performance for behavioral health

Using creative performance as a clinical tool in CATx treatment is not limited to a singular event and CATs may integrate performance-based skills throughout patients’ rehabilitation to reinforce therapeutic gains. Biopsychosocial challenges that may negatively impact behavior such as emotional dysregulation and chronic pain can be addressed through a tactical creativity approach to integrating performance into CATx treatment (11); this can inspire creative expression, increase self-regulation, and improve symptom management (12). Patients can leverage strategies to increase empowerment (54), motivate clinical goal attainment across treatment areas (7), and enhance functioning in diverse environments (11).

The focus of CATx treatment is on the therapeutic process of addressing physiological, psychological, and behavioral goals rather than the artistic products that may transpire from clinical sessions, and this ethos extends to creative performance (55) as experiences are specifically tailored to patients’ treatment plans. For example, a patient who is experiencing anxiety may benefit from first engaging in individual CATx sessions focused on symptom management and desensitization strategies and move towards participating in group sessions that foster connection, increase social trust, and promote comfort in working with others (56). CATx treatment may yield generalizable skills that support creative performance to include grounding and relaxation techniques for managing PTSD-related symptoms (e.g., anxiety, hypervigilance, sleep disturbance) (5, 57), building trust and establishing camaraderie with others through creative engagement in group treatment (19, 58, 59), and gradual exposure to new situations that assist patients in preparing for and coping with change (60). Verbally processing traumatic experiences supports communication goals (18, 61), which can be addressed through creative performance by providing opportunities for patients to speak about the role of CATx treatment in their recovery.

Furthermore, creative performance can help patients gradually decrease avoidance behaviors by engaging in a sequence of carefully titrated exposures where in patients can express creativity in increasingly public settings such as a hospital auditorium, lobby, or outdoor courtyard (11). Within the process of preparing for creative performances, patients learn when and how to employ elements from CATx treatment including emotion regulation techniques, coping skills, and memory strategies so that any activated emotions do not exceed their capacity for affect regulation (62). Breath and movement techniques can help participants leverage their autonomic nervous systems by increasing parasympathetic tone to maintain a physiological state that supports social connection (63). For example, a dance/movement therapist may facilitate a movement experiential following a powerful performance to help patient participants and audience members re-regulate.

### 2.4 Creative performance for social health

Despite the shared culture and camaraderie that accompany military service, military-connected populations can experience entrenched social isolation and loneliness, especially those with PTSD and TBI (64, 65). The impact of PTSD, TBI, and related symptoms on limbic system functioning can yield increased feelings of anxiety and fear (66–68). The relationship between individuals’ mental health and their sense of belonging in, contributing to, and connectedness with their communities has been well-established (69). Social connection has immediate impacts on mental health and health behavior as well as prolonged, cumulative effects on physical health and mortality; therefore, providing a basis to interpret positive socialization as a public health investment (14, 70).

Social health research has found that participation in receptive (e.g., visiting a museum, attending a performance) and creative (e.g., painting, dancing, playing an instrument) cultural activities is associated with improved health, increased levels of life satisfaction, decreased anxiety, and lower depression scores in men and women, with men’s participation having an even stronger positive effect (71, 72). Creative performance has been shown to support social connection in veterans (42, 73) as “the reintroduction of playful and creative outlets provides a healthy outlet for stress, facilitates communication with others, and widens options for socialization” (3, p. 185).

Engagement in CATx treatment may reduce isolation and stigma through meaningful interaction and improve communication with family, peers, and healthcare providers (7, 8). As patients progress in treatment, CATs may encourage them to explore creative performance and/or community arts experiences, as clinically indicated, for consistency of care and continued benefits both in clinical and community settings (32). Participating in group creative performance may promote resilience by connecting through common interests and engaging in teamwork with the shared mission of completing a challenging task (e.g., performance), which aligns with military unit training. As an ongoing preventative practice, patients may continue to yield benefits and strengthen safeguards across personal, familial, and social environments (7, 34).

Additionally, attending events like the CF-CAC may be mutually beneficial for audiences to gain a more thorough understanding of the patients and their rehabilitation processes by witnessing their performances (11). Audiences composed of family members, friends, other patients, healthcare providers, and even the public can position patients as creative community members and help to highlight their strengths rather than limitations, which may result in new insights, empathy, and understanding (10, 34). Healthcare providers many also experience positive outcomes from engaging in or observing creative performances such as decreased professional, personal, and spiritual isolation, which can counteract vicarious trauma and mitigate burnout (74).

### 2.5 Creative performance to support clinical goals

It is common practice for CATs and patients to co-construct clinical goals informed by referral rationale, rehabilitative needs, targeted outcomes, skill areas, and ability levels. When integrating creative performance into treatment, it is necessary to remain goal-oriented to ensure that performance plays an appropriate and supportive role in patients’ overall recovery. Patients’ positioning in the treatment process (e.g., beginning, middle, end) should also be taken into consideration. The integration of CATx treatment and performance supports patients in gaining an increased understanding of how they can leverage innovative and strength-based approaches to address post-injury levels of functioning by focusing on what they *can* do rather than what they *can’t* do (12, 75). **Table 1** provides goal examples and interventions from art therapy, dance/movement therapy, music therapy, behavioral health, and neurocognitive treatment approaches through a lens of creative performance integration.

**Table 1:** Goal- and intervention-informed creative performance integration examples.

Treatment domains	Goal areas	Interventions & descriptions	Creative performance integration examples
Art Therapy	Foster self-expression Strengthen communication	Intervention: Mask making Description: Patients engage in creating masks representing any aspects of their experiences and/or identities (8). Intervention: Box project Description: Patients create a sculpture to communicate public aspects of self on the outside of a box and communicate what is contained and protected in the area inside. This allows for safe communication of trauma, grief, loss, or other personal content otherwise difficult to express (5).	Patients display their artwork as they choose it to be viewed in a supportive setting of family members, fellow patients, and clinic staff. Patients display their artwork and have the opportunity to speak about the intended meaning of symbols and metaphors within their artwork, and/or discuss the art materials or techniques utilized. Patients may use prepared remarks or improvise by speaking in the moment about their artwork.
Dance/ Movement Therapy	Increase embodied self-expression Engage complex motor processes	Intervention: Expressive choreography Description: Patient creates a dance in session to represent their healing journey through expressive movement. This allows patient to externalize physical, psychological, and emotional aspects of their internal experiences (76). Intervention: Movement Sequencing Description: Patient and therapist co-create movement sequences that support motor function which are often set to music. Learning and recall of practiced choreography during performance engages multimodal processing (77).	Patients engage in a dance performance that demonstrates externalization of embodied experiences, which can be used for emotional catharsis and/or as a culminating project to bring closure to dance/movement therapy treatment and encourages continued use of dance/movement practices and performance. Patients are motivated to practice skills needed in the treatment context so they can engage in creative performance that demonstrates memorization of complex dance sequences reflective of their treatment process in a supportive environment.
Music Therapy	Reinforce sense of self through resilience building Improve speech production and respiration	Interventions: Lyric analysis and songwriting Description: Patients analyze lyrics and/or write songs that increase identity awareness, reinforce sense of self, and address vulnerabilities. Resilience-building is supported by acknowledging success and accepting human error (21). Intervention: Therapeutic singing Description: Patients practice enunciation through singing lyrical phrases (series of sequenced notes) and may hold a single tone for a targeted duration to sustain the voice and promote breath support. Singing can enhance patient motivation in other therapies that address related goal areas such as speech/language pathology (7).	Patients present cover songs or original compositions arranged using artistic interpretation that reflects their identities and promotes self-expression. Creative performance provides structure and support for patients to simultaneously experience accomplishments and overcome errors, which can bolster resilience. Patients showcase progress through singing songs (covers or originals) arranged to align with their treatment goals. Creative performance allows patients to apply skills outside of clinical sessions, which may support generalization to other environments (e.g., work, home). Performance may also promote opportunities for care coordination and co-treatment between music therapy other services.
Behavioral Health Neuro-cognitive Health	Decrease anxiety across familial, occupational, and social environments Improve neuroplasticity and synaptic connections between brain regions	Intervention: *In vivo* exposure Description: Patients practice stress management and emotional regulation skills in a supportive environment aimed at increasing confidence and comfort at work and in their community (78). Intervention: Instrument playing Description: Patients experience increased interoperability between regions of the brain, which is particularly important between front and limbic circuits. Enhancement may also occur in motor areas and cerebellar circuits (79).	Patient engagement in creative performance allows for social exposure therapy to occur in titrated amounts thus gradually exposing the patient to a healthy level of stress, which bolsters their confidence and encourages further exposures. Patient engagement in creative performance has been shown to enhance neuroplasticity in both clinical and non-clinical settings. Emerging research indicates neuroplasticity is a key mechanism by which the arts support neuro-cognitive rehabilitation (80) reflected in both functional and structural changes between different brain regions (79).

## 3 Describing the Creative Forces Creative Arts Café

The CF-CAC provides opportunities for patients to creatively demonstrate and express parts of their rehabilitation process in a supportive setting. This aligns with preliminary research on music performance for social transformation, which indicates a shift in patients’ perspective during treatment towards health, resilience, and community (11, 12). In addition to being implemented in clinics and other on-site locations (e.g., hospital lobbies, auditoriums, cafeterias), CATs and community artists have collaborated to host CF-CACs in public spaces (e.g., arts museums, community theatres, libraries) in joint efforts with local military, veteran, and arts organizations (32). Additionally, CF-CACs can be hosted on virtual platforms, which increases accessibility for CATs, patients, and audience members in geographically diverse or isolated locations.

### 3.1 Creative arts therapist role in creative performance

Patient participation in creative performance is voluntary. CATs support patients in making informed decisions whether or not to engage in CF-CACs based on their clinical goals and with consideration to the potential benefits and risks involved. Furthermore, CATs demonstrate through clinical discussion and treatment provision that a patient’s decision to participate in creative performance does not affect the therapeutic relationship nor impact their access to treatment. When patients choose to engage in creative performance, CATs and patients collectively determine how much assistance is needed. The CAT’s level of engagement exists on a spectrum from minimal (e.g., audience member) to maximal (e.g., co-performing with patients). Examples include being visible to the patient to provide performance cuing such as mouthing lyrics or modeling choreography. Additionally, CATs may musically accompany patients (11, 81), dance with them (59), or serve as a gallery guide when patient’s artwork is presented (34).

Preparatory groups provide necessary rehearsal time wherein patients can reinforce strategies from CATx treatment to employ during performances as needed. Examples include locating a visual focal point to sustain concentration (82), identifying physical posturing and positioning optimal for spatial orientation and grounding (83), and practicing intra-and interpersonal entrainment for emotional regulation and connection to the performance and the audience (84). Applying principles of graded exposure that move from least anxiety provoking (e.g., identifying a song to sing) to most anxiety provoking (e.g., singing the song in public) while rehearsing in CATx sessions are a necessary step in performance preparation to help mitigate performance anxiety (85).

Intentional practice also promotes autonomy, supports socialization, and bolsters camaraderie for individual and group performers. An essential part of rehearsals and creative performances is that patients can navigate challenging experiences in a supportive environment wherein they experience applying skills from CATx treatment to performance and generalize skills from performance to daily life situations, which can help to optimize functioning in non-performative (e.g., familial, social, occupational) contexts (11). CATs and patients work together to determine the level of support that a patient may require during performance and decide what products from their CATx treatment they will perform or exhibit (11, 86).

These preparatory process are critical for building autonomy and empower engagement in arts-based exhibitions and interactive performances (87). CATs should remain alert throughout practice sessions and creative performance experiences to assess patient needs, identify emergent issues, and support patients’ clinical goals and treatment processes. While some performance objectives may more obviously align with patients’ CATx goals, it is also beneficial to identify overlap with treatment plans of other clinical services they are receiving.

### 3.2 Interdisciplinary care coordination for creative performance

In interdisciplinary settings, CATs work closely with rehabilitation and behavioral health providers wherein the care team works collaboratively to address patients’ rehabilitative needs by creating customized care plans, providing goal-based treatment, and tracking clinical progress. This environment allows for consultation and co-treatment between team members, which is paramount for overall care coordination and provides multi-level support for patients who engage in creative performance. Therefore, it is highly beneficial for CATs to communicate, consult, and/or co-treat with team members to provide patients with a high level of care and support creative performance integration. Provider teams can formulate treatment plans that consider patient safety and identify potential for negative impact on rehabilitative progress.

Communication can take place directly between CATs and other providers, during team meetings, or through written correspondence (e.g., encrypted email, secure messaging). The benefits of interdisciplinary team coordination are three-fold: 1) patients can demonstrate gains of clinical goals across multiple treatments; 2) patients are supported by a team of providers, which increases patient safety and mitigates risk; and 3) interdisciplinary team members deepen their understanding of patients’ progress and each other’s work through monitoring patient responses to various therapies, which can strengthen provider rapport and inform cross-discipline referrals.

### 3.3 Detailing the CF-CAC process

**Table 2** provides a CF-CAC process description, highlights specific components, and provides guidance of how to apply creative performance using this model.

**Table 2:** CF-CAC components and process description.

Component	Process description
Preparing patients from the clinic to the stage	The therapeutic relationship: Supporting patients on the clinic to community continuum. i. Informed consent ensures patient safety, autonomy, opportunities for decision making, and HIPAA compliance. CATs maintain records in a secure location [Sample form provided as **Supplementary Material 1**]. ii. Risk mitigation of creative performance includes communicating potential benefits and risks, pre-and post-processing, continued evaluation, and support during the CF-CAC. CATs assess each patient’s unique capacity for risk-taking before integrating creative performance into treatment. iii. Preparatory tasks (e.g., CF-CAC prep groups) assess patients’ capacity for creative performance and address potential reservations about participation.
Integration of patient goals and creative performance	Creative performance in CATx treatment: Patient goals are the primary focus. i. CATs review treatment goals with patients prior to creative performance to identify how participation in CF-CAC supports patients’ CATx treatment. ii. Patients have the option to prepare any information they want to share with the audience about how creative performance supports their clinical goals across treatment areas. iii. Co-performance between patients and CATs and/or other providers is accommodated, as appropriate and clinically indicated.
Logistical considerations of event space, safety, accessibility, and compliance Advertisement and promotion	Creative performance venue: Ensuring a safe, accessible, compliant, and accommodating environment. i. Ensure that the location is ADA compliant, accessible, and appropriate for performance. ii. Identify nearest power supply, restrooms, safety exits, and formulate spatial configuration to meet patient, audience, and environmental needs. iii. Set up stage and seating prior to the event so patient performers can familiarize with the space. Creative performance promotion: Engaged audiences build community connections. i. Create printed and digital programs that contain event information (e.g., time/date, agenda, performances) prior to the event. Programs can also serve as a memento of the event for patients and attendees. With permission, programs may include performers’ name and title of performance, featured guest artists or organizations, and any other relevant event information [Sample program provided as **Supplementary Material 2**]. • Distribute event flyers in highly trafficked areas around the clinic or hospital/facility-at-large approximately one week prior to the CF-CAC*. • Email a digital copy of the flyer to the site email distribution list a week in advance and again 1–2 days before the event for increased awareness*. *Permissions may be required to distribute promotional material in medical facilities.
Step-by-step CF-CAC agenda Post-performance after-actions and documentation	CF-CAC agenda: Event organization is essential to prepare patients for the performance flow. i. Two hours prior to CF-CAC: Check in with the patient performers, ensure that all required consents/waivers are signed, venue set-up is complete, and that patients have the necessary materials (music, instruments, artwork, presentation notes) for their performances. ii. One hour prior to CF-CAC: Performers convene in a designated space to review the event agenda (when they are performing, how much time they have) and rehearse their pieces. iii. 30 minutes prior to CF-CAC: Any final preparatory requirements are completed and performers transition from rehearsal space to performance venue. iv. 10 minutes prior to CF-CAC: Greet audience members, distribute programs, and announce the event will commence shortly. v. Show time: A designated person (e.g., CAT, clinic leadership) provides opening remarks, introduces the ‘emcee’ (e.g., patient, staff member), and the CF-CAC begins and follows the prepared agenda. vi. CF-CAC closure: A designated person provides closing remarks acknowledging the patient performers, audience members, any special guests (e.g., community collaborator), venue/site support, and funding sources. vii. Immediately following CF-CAC: Check in with all patient performers to assess any need for immediate processing or follow-up support. Breakdown the performance venue. Process performance experiences and discuss the impact on goals and treatment plan. i. Address any identified immediate patient needs before they leave. If patients are stable, continued performance processing can occur during the patient’s next CATx appointment. ii. Document the impact of creative performance on treatment goals in patients’ electronic medical records in accordance with site documentation policy. • Recommended documentation may include patient report of the performance experience; therapist observation of patients’ performance experience; impact of performance on patients’ goal progress; and patient safety assessment. [Sample documentation template provided as **Supplementary Material 3**].

### 3.4 Potential contraindications and recommended safeguards

The arts are a powerful sensory stimuli; thus, there is potential for creative performance to elicit strong emotional reactions in both patient performers and audience members. Therefore, potential contraindications must be considered prior to patient participation. For example, performance could be re-traumatizing for patients who cannot self-regulate, experience high levels of social or performance anxiety, or exhibit adverse responses to vulnerability (11, 81). In such cases, creative performance in CF-CACs may not be clinically indicated. As a fundamental priority of clinical work, patient safety is extensively considered in creating and refining CF-CAC processes and procedures to include safeguards such as ample time for pre-performance preparation, provider support during performances, and post-performance processing.

Given the prevalence of suicide among military-connected populations, CATs and interdisciplinary team members integrate these safeguards across planning, preparation, and implementation of CF-CAC events, as detailed in **Table 2**, to ensure safety of the patient performers, the audience members, and the overall performance environment. Safety assessment is implemented throughout the CF-CAC process, and patients’ creative performance experiences are documented in electronic medical records (**Table 2**; **Supplementary Material 3**). Additionally, if concerns arise from performance participation, the patient’s behavioral health provider is notified to follow-up with the patient, further process the experience, and ensure their safety.

## 4 Discussion

CATx treatment approaches that include creative performance, such as the CF-CAC model, support interdisciplinary treatment optimization of military-connected individuals with TBI, PTSD, and related health issues. Integrating performance into treatment has demonstrated clinical beneficial and is also not without controversy. In the context of CATx treatment, there is a clear focus on the therapeutic process, rather than the creative product; therefore, where there may be unintended negative consequences of self-disclosure, CATs may recommend that patients not participate in performance. Other perspectives include the potential for performance to confound essential elements of therapy such as of privacy, confidentiality, and the increased risk of stressful situations for patients (88). Conversely, some CATs who operate within community models have posited that performance has the capacity for enriching patients’ health and well-being (89). This is achieved by assisting patients in negotiating and navigating their positionality between the institution and the community-at-large (75) and encourages therapists to shift focus on when, rather than if, performance is considered an appropriate part of treatment (90).

### 4.1 Considerations for virtual CF-CAC

During the COVID-19 global pandemic, healthcare providers pivoted to telehealth practices to conduct clinical sessions on virtual platforms, which included CATx treatment (29). Deriving learning from pre-pandemic telehealth practices, CATs adapted CF-CAC to online spaces using government-approved platforms that accommodated creative performance in support of patients’ clinical goals and community engagement efforts (91). Similar to telehealth, which maintained relevance as a viable treatment option in healthcare, CF CATs continue to implement the virtual CF-CAC model to date. In addition to the benefits of in-person creative performance, other therapeutic goals may be attained through virtual facilitation. For example, participation may be more accessible in virtual environments for patient performers and their families, and they may find comfort in participating from their homes (6). CATs can also leverage technological disruptions (e.g., audio/visual latency, competing sounds, connectivity issues) as opportunities to assist patients in expanding thresholds of frustration tolerance and practicing emotional regulation strategies (29).

The virtual CF-CAC model follows the same core processes as the in-person model and supports patients’ needs by offering alternative outlets for socialization. The virtual space offered opportunities for co-hosting events thus enabling patients, families, and providers in different locations to collaborate with their counterparts at networked facilities within DoD and VA healthcare systems. The virtual CF-CAC model created space for service members, veterans, their families, and provider teams to interact while building a sense of community through the arts, which participants reported was enriching, particularly during a prolonged period of widespread isolation.

### 4.2 Limitations and future directions

A limitation of the CF-CAC model is the lack of standardized evaluation on the impact and outcomes of creative performance engagement. It is customary for CATs to solicit feedback from patient performers, audience members, providers, site leadership, and community entities about their experiences with creative performance, and the use of validated self-report measures can increase understanding of participant outcomes and indicate areas of improvement to optimize the CF-CAC process. Future studies of creative performance could benefit from data collection, for example, a post-event survey administered to performers and audience members that includes information such as role (e.g., patient, family, provider/staff), purpose of attendance (e.g., performer, audience, support role), number of events attended or performed, and reported benefits of engagement.

Validated self-report measures such as the UCLA-3 maybe helpful as an outcome measure of connectedness (92) to inform understanding of the impact of loneliness and social isolation (93), both of which are common factors in military-connected populations specifically with TBI, PSTD, and related sequelae. Other factors aligned with creative performance that could be measured include creativity (94, 95), meaning making in the contexts of self-efficacy, mastery, positive reinterpretation, and acceptance (96), and enjoyment related to engagement, challenge/improvement, and competence (97). In clinical settings where CATs work with behavioral health professionals, outcome measures may be administered as part of integrative treatment. It is recommended that CATs also employ observational assessments of CF-CAC participants to determine biopsychosocial-spiritual impact (98) and identify immediate outcomes that can inform long-term sustainable gains of continued engagement in creative performance. Additionally, consulting with community entities that participate in CF-CAC events to glean their experience and identify areas for improvement is recommended.

Another important consideration is to evaluate the sustainability of integrating creative performance into healthcare systems by consulting primary stakeholders (e.g., patients, audience members, healthcare providers, leadership). This should include decision-makers at the highest levels to understand what they identify as most important and impactful to the overarching rehabilitative landscape. Investment in creative performance from both healthcare consumers and institutional leadership can help shape implementation and evaluation processes, which are necessary to drive continued development (99). Furthermore, having infrastructure support including dedicated time for CATs’ coordination efforts, space for events, staff support, and institutional buy-in are critical components of ensuring that the CF-CAC model is sustainable.

A co-author of this paper who served as a Navy psychiatrist and directed a military clinic, shared his perspective on the impact of the CF-CAC for patients with TBI and PTSD and overall clinic morale:

I was first exposed to the CF-CAC when a music therapist began working at a specialty TBI clinic at a leading military treatment facility. In learning about and observing music therapy treatment of patients with TBI and PTSD, it was clear that creative arts therapies and performance were bridging approaches, as they address and reinforce emotional expression, memory and attentional sustainment, neural circuitry and cognition, visuospatial processing, and fine motor control. Behaviorally, aspects of performance directly address fear of public spaces, and the partnering aspects of therapy create opportunities for peer support and socialization. From a caregiver support perspective, families and staff benefit alongside the patients. In a military environment where the formality and hierarchy can sometimes inhibit deeper human connections, the CF-CAC was a grounding tool that improved both the quality of effort put into recovery and the quality care that was rendered (3).

In addition to supporting patients’ creative performance processes and gaining feedback from performers and audience members, it is recommended that CATs reflect on their own experiences and observations. Collectively, these mechanisms promote increased awareness for developing informed practices to best serve patients through creative performance (75). This can be accomplished through professional activities such as completing appropriate documentation in the electronic medical record (**Table 2**; **Supplementary Material 3**), maintaining a document of de-identified clinical observations from creative performance experiences, and engaging in peer supervision with fellow providers. The CF-CAC model provides a collaborative, strength-based, and experiential creative environment that can amplify patients’ rehabilitative outcomes and boost morale of providers and the overall clinic culture (4). Creative performance provides opportunities for patients to experience new levels of agency and autonomy in their treatment, for clinicians to observe patients’ post-traumatic growth, and for both providers and patients to identify future-focused directions for treatment conceptualization, implementation, and optimization (11).

## Conclusion

The integration of creative arts therapies, interdisciplinary care, and creative performance is an innovative approach aimed at improving treatment outcomes for military-connected patients with TBI, PTSD, and related health concerns. The inclusion of creative performance into treatment benefits the patients, supports providers, boosts clinic morale, and strengthens familial and community connections. Creative performance can serve as a catalyst for increased healing and extended outreach opportunities at micro, mezzo, and macro levels as it supports patient well-being by fortifying connections within their rehabilitation processes and providers as well as to their families, peers, and communities-at-large.

## Author disclaimer

The opinions and assertions expressed herein are those of the authors and do not necessarily reflect the official policy or position of the Uniformed Services University, the Defense Health Agency, the Department of Defense, nor the U.S Government. The opinions and assertions expressed herein are those of the authors and do not necessarily reflect the official policy or position of the Henry M. Jackson Foundation for the Advancement of Military Medicine, Inc., or the National Endowment for the Arts. Mention of trade names, commercial products, or organizations does not imply endorsement by the U.S. Government.
