# Supplementary figures and images for: Creative Forces® Creative Arts Café: a theory-based creative performance framework for military-connected populations with traumatic brain injury and posttraumatic stress disorder
Source: Front Psychiatry. 2026 Mar 26;17:1734583. doi: 10.3389/fpsyt.2026.1734583 (PMC13062902; doi:10.3389/fpsyt.2026.1734583)

**SUPPLEMENTAL MATERIAL 1: Sample Informed Consent Form**

**
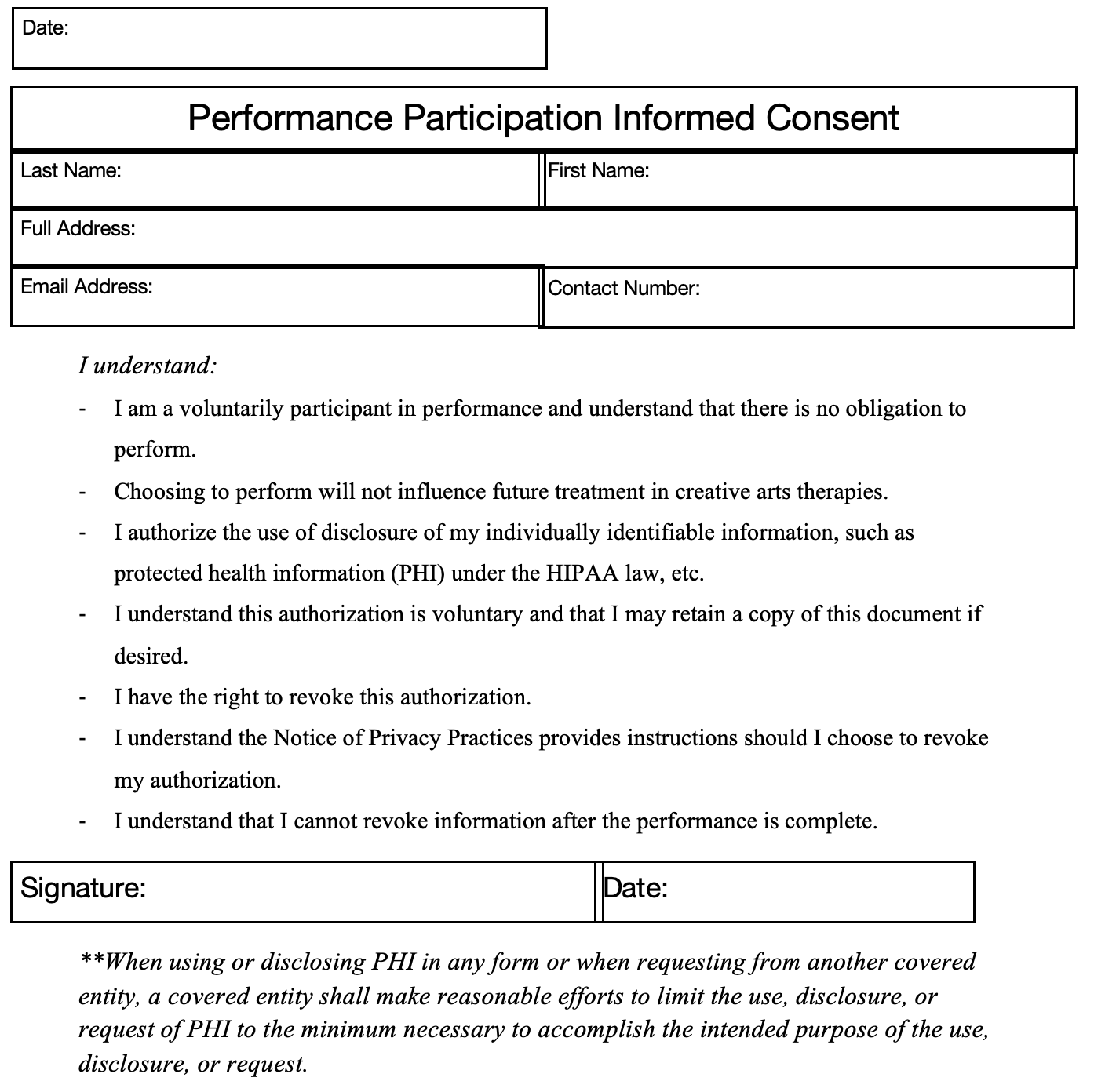
**

Supplement: Supplementary file 1 [file Supplementaryfile1.docx]

**SUPPLEMENTAL MATERIAL 2: Sample CF-CAC Program** (based on a 60-minute event)
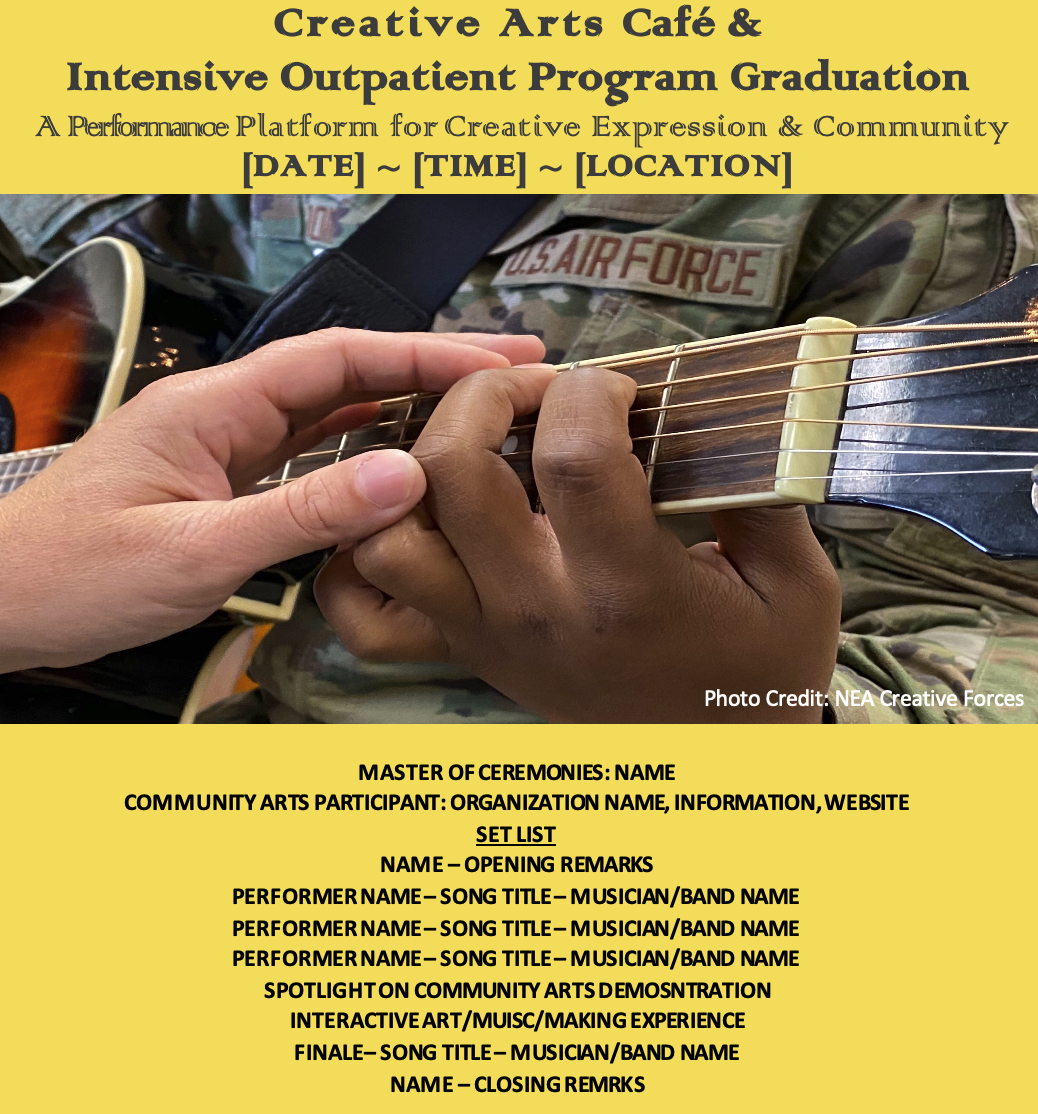

Supplement: Supplementary file 2 [file Supplementaryfile2.docx]
