# Supplementary material for: Creative Forces® Creative Arts Café: a theory-based creative performance framework for military-connected populations with traumatic brain injury and posttraumatic stress disorder
Source: Front Psychiatry. 2026 Mar 26;17:1734583. doi: 10.3389/fpsyt.2026.1734583 (PMC13062902; doi:10.3389/fpsyt.2026.1734583)
Supplement: Supplementary file 3 [file Supplementaryfile3.docx]

**SUPPLEMENTAL MATERIAL 3: Sample Creative Performance Documentation**

**In-person CF-CAC note template:**

The patient consented to and engaged in creative performance via a Creative Arts Café. Creative performance aims to support positive social interaction through arts engagement on a clinic to community continuum. Participation included [#] fellow patient/peer performers and approximately [#] audience members. The patient [did/did not] introduce their piece, [did/did not] explain the impact of creative arts therapies on their rehabilitation and engaged in performance through [insert creative arts medium]. Creative performance [advanced/didn’t advance] the patient’s treatment goals of [insert goals] as evidenced by [insert rationale]. The patient reported that the performance experience [patient feedback]. Creative arts therapist observation indicates [therapist feedback]. Post-performance processing will be offered to the patient at their next appointment on [date/time]. Patient [is/is not] recommended to continue engaging in creative performance. [Additional comments].

**Virtual CF-CAC note template:**

The patient consented to and engaged in virtual creative performance via a virtual Creative Arts Café hosted on [indicate virtual platform]. Patient’s physical location was confirmed [address, city, state]. In the event of an emergency during the performance, the patient’s phone number and emergency contact information were confirmed [contact information]. Virtual creative performance aims to support positive social interaction through arts engagement on a clinic to community continuum. Participation included [#] fellow patient/peer performers and approximately [#] audience members. The patient [did/did not] introduce their piece, [did/did not] explain the impact of creative arts therapies on their rehabilitation and engaged in virtual performance through [insert creative arts medium]. Virtual creative performance [advanced/didn’t advance] the patient’s treatment goals of [insert goals] as evidenced by [insert rationale]. The patient reported that the virtual performance experience [insert patient feedback]. Creative arts therapist observation indicates [insert therapist feedback]. The patient [did/did not] report experiencing technical difficulty during the virtual performance. Post-performance processing will be offered to the patient at their next appointment on [insert date/time]. Patient [is/is not] recommended to continue engaging in virtual creative performance. [Additional comments].
